# Orexin receptor agonist Yan 7874 is a weak agonist of orexin/hypocretin receptors and shows orexin receptor-independent cytotoxicity

**DOI:** 10.1371/journal.pone.0178526

**Published:** 2017-06-02

**Authors:** Ainoleena Turku, Maiju K. Rinne, Gustav Boije af Gennäs, Henri Xhaard, Dan Lindholm, Jyrki P. Kukkonen

**Affiliations:** 1 Biochemistry and Cell Biology, Department of Veterinary Biosciences, University of Helsinki, Helsinki, Finland; 2 Division of Pharmaceutical Chemistry and Technology, Faculty of Pharmacy, University of Helsinki, Helsinki, Finland; 3 Department of Biochemistry and Developmental Biology, Medicum, Faculty of Medicine, University of Helsinki, Helsinki, Finland; 4 Minerva Foundation Institute for Medical Research, Helsinki, Finland; Universite de Rouen, FRANCE

## Abstract

Two promising lead structures of small molecular orexin receptor agonist have been reported, but without detailed analyses of the pharmacological properties. One of them, 1-(3,4-dichlorophenyl)-2-[2-imino-3-(4-methylbenzyl)-2,3-dihydro-1*H*-benzo[*d*]imidazol-1-yl]ethan-1-ol (Yan 7874), is commercially available, and we set out to analyze its properties. As test system we utilized human OX_1_ and OX_2_ orexin receptor-expressing Chinese hamster ovary (CHO) K1 cells as well as control CHO-K1 and neuro-2a neuroblastoma cells. G_q_-coupling was assessed by measurement of intracellular Ca^2+^ and phospholipase C activity, and the coupling to G_i_ and G_s_ by adenylyl cyclase inhibition and stimulation, respectively. At concentrations above 1 μM, strong Ca^2+^ and low phospholipase C responses to Yan 7874 were observed in both OX_1_- and OX_2_-expressing cells. However, a major fraction of the response was not mediated by orexin receptors, as determined utilizing the non-selective orexin receptor antagonist *N*-biphenyl-2-yl-1-{[(1-methyl-1*H*-benzimidazol-2-yl)sulfanyl]acetyl}-L-prolinamide (TCS 1102) as well as control CHO-K1 cells. Yan 7874 did not produce any specific adenylyl cyclase response. Some experiments suggested an effect on cell viability by Yan 7874, and we thus analyzed this. Within a few hours of exposure, Yan 7874 markedly changed cell morphology (shrunken, rich in vacuoles), reduced growth, promoted cell detachment, and induced necrotic cell death. The effect was equal in cells expressing orexin receptors or not. Thus, Yan 7874 is a weak partial agonist of orexin receptors. It also displays strong off-target effects in the same concentration range, culminating in necrotic cell demise. This makes Yan 7874 unsuitable as orexin receptor agonist.

## Introduction

Orexin receptor stimulation is expected to have important pharmacological uses, most obviously in symptomatic treatment of narcolepsy ([1–3]; reviewed in [4–7]), and maybe other sleep and vigilance disorders. Orexin receptor stimulation is able to induce programmed cell death in different types of cancer cells [8, 9], and thus might offer a novel way of cancer treatment. Orexin signalling is also involved in a wide range of other functions such as addiction, stress response and metabolic regulation [10, 11], and it is feasible that therapeutic areas are born out of these.

**Fig 1:**
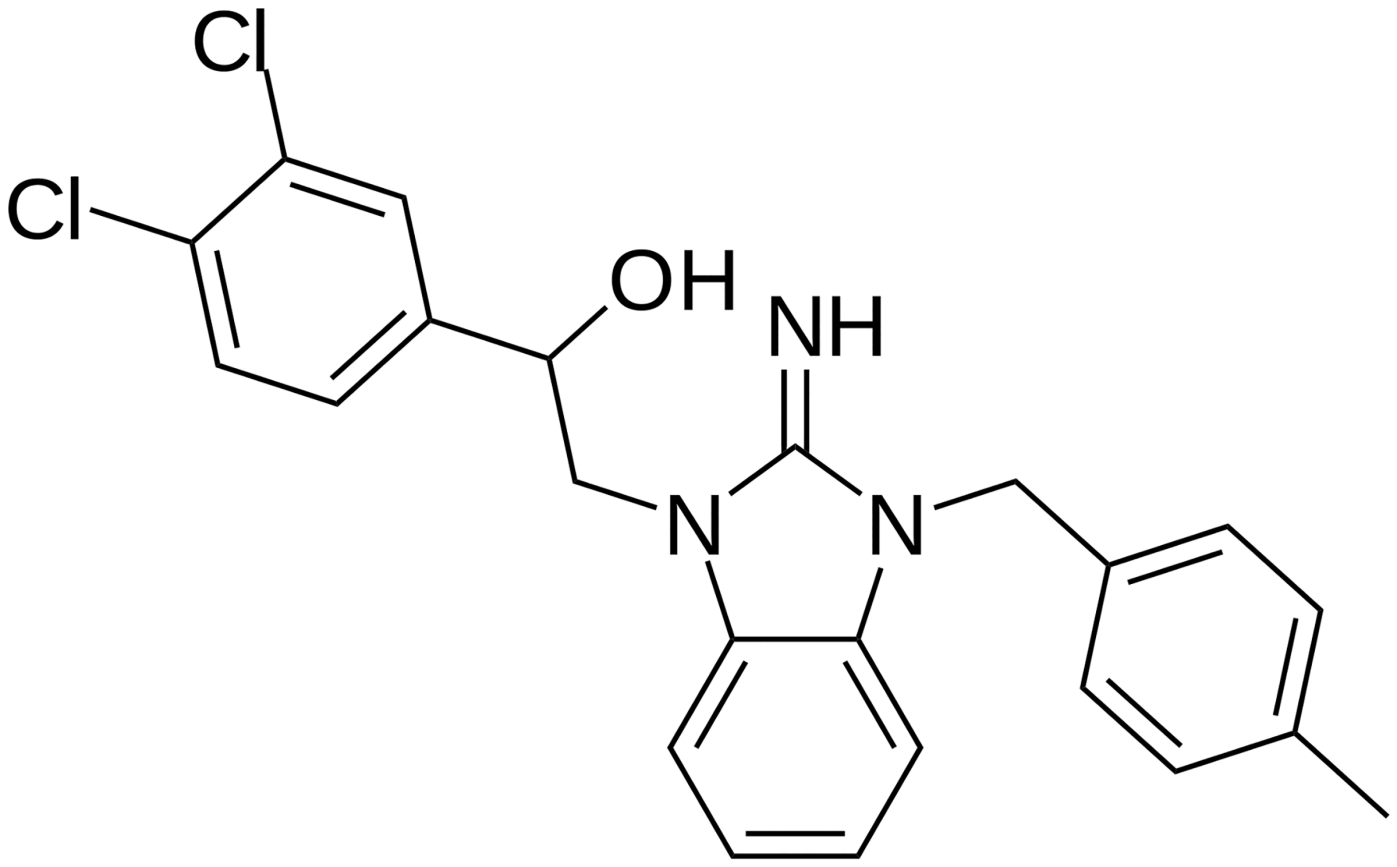
Yan 7874 structure.

However, there were no small molecule agonist ligands for years; the only known agonist ligands for orexin receptors were the native orexin peptides (orexin-A and -B) or synthetic variants, which, as peptides, likely do not show advantageous pharmacodynamic properties. While tens of lead structures of orexin receptor antagonists have been developed during the last 17 years [12–14], no corresponding development has been reported on the agonist field, except for two promising series of small molecular orexin receptor agonists. The first series has so far only been published through a patent [15], while the second series was published in a peer-reviewed journal [16]. Common for these studies is that very few details of the pharmacological properties of even the lead structures have been published, and for what is published, the assay details etc. are not revealed. To date, the original publications [15, 16] remain as sole sources of information; lack of a commercial source for these compounds is the likely explanation for the lack of other, independent studies. Nevertheless, these compounds are of high interest, not only for the drug use but also for use as research tools. We recently found that one of the compounds, 1-(3,4-dichlorophenyl)-2-[2-imino-3-(4-methylbenzyl)-2,3-dihydro-1*H*-benzo[*d*]imidazol-1-yl]ethan-1-ol (Yan 7874), the lead structure of the first series (Fig 1), is commercially available. We thus set out to examine this ligand for a set of orexin receptor responses representing different G-protein outputs, utilizing Chinese hamster ovary K1 (CHO-K1) cells expressing recombinant human orexin receptors as the model.

## Materials and methods

### Cell culture and media

CHO-K1 cells expressing human OX_1_ and OX_2_ receptors cells (CHO-hOX_1_ and CHO-hOX_2_, respectively; [17, 18]) as well as ctrl CHO-K1 cells (not expressing orexin receptors; ctrl CHO cells) were cultured in Ham’s F12 medium (Gibco/Life Technologies, Paisley, UK) + supplements, and neuro-2a cells in Dulbecco’s modified Eagle’s medium (Gibco/Life Technologies) + supplements on plastic cell culture dishes (56 cm^2^ bottom area; Greiner Bio-One GmbH, Frickenhausen, Germany) as described in [19, 20]. Different types of multi-well plates were used for the assays: black, clear-bottom half-area Cellstar μClear 96-well cell culture plates (Greiner Bio-One GmbH; Frickenhausen, Germany) for Ca^2+^ measurements; black, clear-bottom Cellstar μClear 96-well cell culture plates (Greiner) for cell death assays; and clear Cellstar 48-well cell culture plates (Greiner) for phospholipase C (PLC) and adenylyl cyclase (AC) assays. All multi-well plates were coated with polyethyleneimine (25 μg/mL for 1 hour at 37°C; Sigma-Aldrich, St. Louis, MO, USA). Cells were for some AC experiments pretreated for 20 h with cholera toxin (CTx; 1000 ng/mL) or for 36 h with pertussis toxin (PTx; 300 ng/mL).

Hepes-buffered medium (HBM) was used as the basic experimental medium. It was composed of 137 mM NaCl, 5 mM KCl, 1.2 mM MgCl_2_, 0.44 mM KH_2_PO_4_, 4.2 mM NaHCO_3_, 1 mM CaCl_2_, 10 mM glucose, 20 mM HEPES and 0.1% (w/v) stripped bovine serum albumin [21], and adjusted to pH 7.4 with NaOH.

### Ca^2+^ elevation

**Fig 2:**
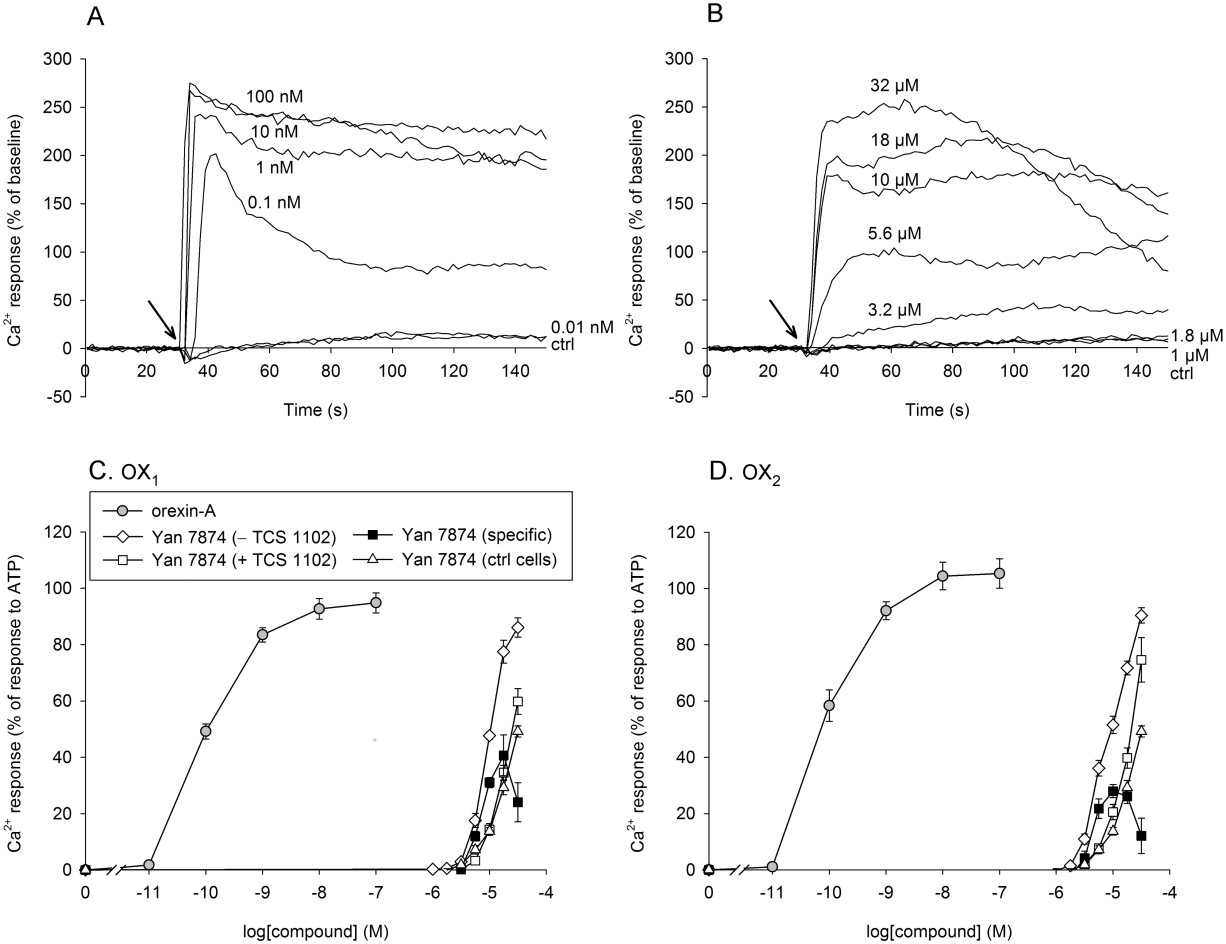
Ca^2+^ elevation in CHO cells. (A–B) Representative traces for orexin-A (A) and Yan 7874 (B) responses in CHO-hOX_2_ cells. The responses are presented as fluorescence increase above the baseline. (C–D) Concentration-response curves for orexin-A and Yan 7874 responses in CHO-hOX_1_ (C) and -hOX_2_ (D) cells as well as control CHO cells not expressing any orexin receptors ["Yan 7874 (ctrl cells)"] (C and D). The responses are presented as normalized to the response to 100 μM ATP to allow comparison of the Yan 7874 response magnitudes in orexin receptor-expressing and non-expressing (ctrl) cells. The responses were normalized to the ATP response (100%) separately for each independent sample before averaging. Yan 7874 responses are shown both as such ["Yan 7874 (–TCS 1102)"] as well as in the presence of 10 μM TCS 1102 ["Yan 7874 (+ TCS 1102)"]; "Yan 7874 (specific)" indicates subtraction of the latter from the former. *N* = 6.

Ca^2+^ elevations were measured utilizing the method described in our earlier study [22]. The cells, 1.5×10^4^ per well, were plated on black, clear bottom half-area 96-well plates. Twenty-four hours later, cell culture medium was removed and the cells were loaded with the loading solution composed of FLIPR Calcium 4 Assay Kit (Molecular Devices, Sunnyvale, CA, USA) dissolved in and diluted with HBM + 1 mM probenecid, for 60 min at 37°C. Then, the plate was placed in a FlexStation 3 fluorescence plate reader (Molecular Devices) and the intracellular Ca^2+^ levels were measured as fluorescence changes (excitation at 485 nm, emission at 525 nm) at 37°C. A recording was made approximately every 1.3 s. Each well was measured for 150 s with 30 s of baseline before stimulation (see Fig 2A and 2B). Antagonists were manually added in the wells during the probe incubation (30 min before the measurement).

### Phospholipase C activity

PLC activity was measured utilizing the method described in our earlier study [23]. The cells, 2.6×10^4^ per well, were plated on clear 48-well plates. After 24 h, they were labelled with 3 μCi/mL [^3^H]-inositol for 20 h. The medium was removed, and the cells were incubated in HBM supplemented with 10 mM LiCl for 30 min at 37°C; also the possible inhibitors [*N*-biphenyl-2-yl-1-{[(1-methyl-1*H*-benzimidazol-2-yl)sulfanyl]acetyl}-L-prolinamide (TCS 1102) or L-threonine,(3*R*)-*N*-acetyl-3-hydroxy-L-leucyl-(a*R*)-a-hydroxybenzenepropanoyl-2,3-idehydro-*N*-methylalanyl-L-alanyl-*N*-methyl-L-alanyl-(3*R*)-3-[[(2*S*,3*R*)-3-hydroxy-4-methyl-1-oxo-2-[(1-oxopropyl)amino]pentyl]oxy]-L-leucyl-*N*,*O*-dimethyl-,(7→1)-lactone (9CI) (UBO-QIC)] were included in this incubation. They were then stimulated with orexin-A or Yan 7874 for 10 or 30 min, after which the medium was rapidly removed and the reactions stopped by adding ice-cold 0.4 M perchloric acid and freezing. The samples were thawed and neutralized with 0.36 M KOH + 0.3 M KHCO_3_, and the insoluble fragments spun down. The total inositol phosphate fraction of the supernatants was isolated by anion-exchange chromatography, and the radioactivity determined by scintillation counting (HiSafe 3 scintillation cocktail and Wallac 1415 liquid scintillation counter; PerkinElmer).

### Adenylyl cyclase activity

AC activity was determined utilizing the method described in our earlier study [23]. The cells, 2.4–3.0×10^4^ per well, were plated on 48-well plates. The cells were treated with PTx after 8 h or with CTx after 24 h. Forty-eight hours after the plating, the cells were labelled with 5 μCi/mL [^3^H]-adenine for 2 h. After the labelling, the cells were washed once with PBS. HBM, supplemented with 500 μM 3-isobutyl-1-methylxanthine (a cyclic nucleotide PDE inhibitor) and 3 μM 2-(1-[3-dimethylaminopropyl]-1*H*-indol-3-yl)-3-(1*H*-indol-3-yl)-maleimide (GF109203X; a protein kinase C inhibitor), was added to the cells. The cells were incubated for 30 min at 37°C before adding the stimulants [forskolin ± orexin-A, Yan 7874 or (2*R*,6a*S*,12a*S*)-1,2,12,12a-tetrahydro-8,9-dimethoxy-2-(1-methylethenyl)-[1]benzopyrano[3,4-*b*]furo[2,3-*h*][1]benzopyran-6(6a*H*)-one (rotenone)]. After an additional 10-min incubation at 37°C, the medium was discarded and the reactions terminated with ice-cold perchloric acid and rapid freezing. After thawing, the insoluble fragments were spun down, and the [^3^H]-ATP + [^3^H]-ADP and [^3^H]-cAMP fractions were isolated by sequential Dowex-alumina chromatography. Radioactivity was determined from the fractions with scintillation counting as above. The conversion of [^3^H]-ATP to [^3^H]-cAMP was calculated as percentage of the total eluted [^3^H]-ATP + [^3^H]-ADP.

### Cell viability and death

Cells were seeded on black, clear bottom 96-well plates (0.75–1.5×10^4^ per well, depending on the experiment run time) for the plate reader assay. The cells were cultured overnight and treated the following day. Bright-field microscopic observation was carried out once every 24 h up to 72 h. Quantitation of the staining was done for cell treated for 24 h, since a robust cell death was seen already then.

Phase contrast microscopy was done on Olympus CKX41 microscope with attached Canon EOS 600D digital camera; this assesses only the morphological features of the cells. For fluorescent microscopy, the cells were stained with Hoechst 33342 (Hoechst; 10 μM), propidium iodide (PI; 1 μM) and fluorescein diacetate (FDA; 1 μM) for 20–30 min at 37°C. Hoechst stains all nuclei, but the morphology of the nucleus can distinguish normal or necrotic cells (large, diffuse) from apoptotic cells (condensed, possibly fragmented). PI can only permeate damaged plasma membranes and can thus only stain the nuclei of necrotic cells. FDA (non-fluorescent) is hydrolyzed by non-specific esterases in the cytoplasm of viable cells, releasing fluorescent fluorescein, which is retained only by the cells of intact membranes; thus FDA is a measure of both cell viability and membrane integrity. Fluorescent microscopy was performed on Nikon TE2000 fluorescence microscope (Nikon, Tokyo, Japan) with 20×/0.75 air objective and the images acquired by an Andor iXon 885 electron-multiplying charge-coupled device camera (Andor Technology Ltd., Belfast, UK) under the control of Nikon NIS Elements AR software.

Plate reader was used for quantitative assessment of the cell numbers, cell viability and necrosis. The cells were stained as above, either directly in the culture medium or the culture medium was first removed and replaced with HBM. While the former method seemed to result in large FDA background, the latter may reduce the total cell number since some detached cells are removed. After the incubation as above, the fluorescence was read using FlexStation 3 at the wavelengths 352 nm/455 nm (Hoechst), 480 nm/525 nm (FDA) and 538/617 nm (PI). Please observe that this assay does not assess apoptotic cell death, since the plate reader only measures the gross fluorescence and not its pattern, and thus the different types of Hoechst-stained nuclei are not distinguished. Hoechst fluorescence thus only gives the total cell number in this assay.

### Materials

Human orexin-A was from NeoMPS (Strasbourg, France), GF109203X, TCS 1102 and PTx from Tocris Bioscience (Bristol, UK), and CHX, CTx, FDA, forskolin, 3-isobutyl-1-methylxanthine, PI and rotenone from Sigma-Aldrich (St. Louis, MO, USA). *Myo*-[2-^3^H]-inositol (PT6-271) and [2,8-^3^H]-adenine were from PerkinElmer Life and Analytical Sciences (Waltham, MA, USA). Hoechst was from Molecular Probes/Life Technologies (Carlsbad, CA, USA) and Yan 7874 from ChemBridge Co. (San Diego, CA, USA).

### Data analysis

**Fig 3:**
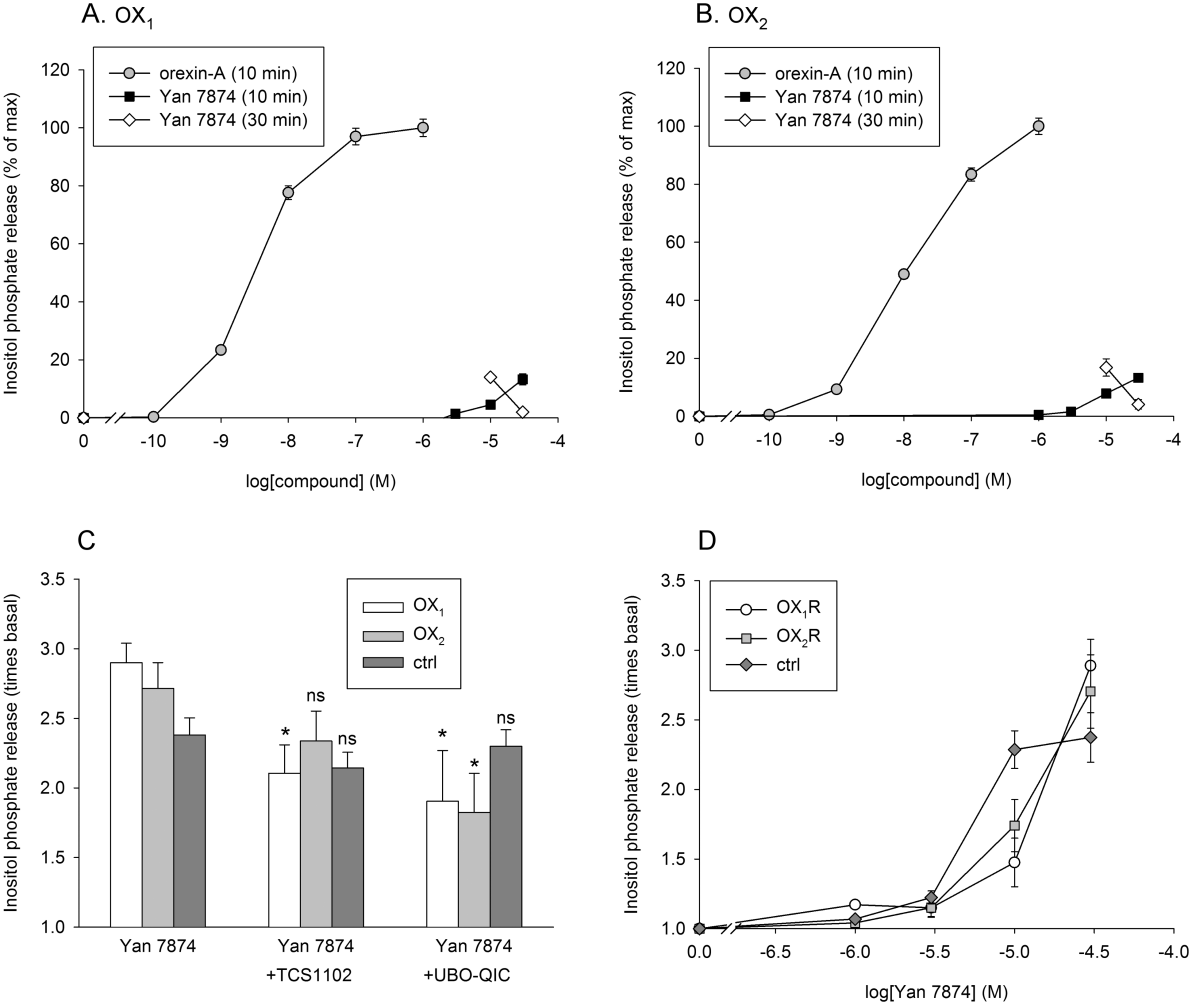
PLC activity in CHO cells. (A–B) Orexin-A and Yan 7874 concentration-response curves in OX_1_- (A) and OX_2_-expressing (B) cells normalized to the maximum response at the corresponding stimulation time (10 or 30 min) as determined by curve-fitting. The responses were normalized to the orexin-A response (100%) separately for each independent sample before averaging. (C) Inhibition of Yan 7874 response (30 μM, 10 min) by the orexin receptor antagonist TCS 1102 (10 μM) and the G_q_ antagonist UBO-QIC (1 μM). The comparisons are to the corresponding control in the absence of any inhibitor for each cell type. (D) Responses in OX_1_ and OX_2_ cells and ctrl cells expressing no orexin receptors. *N* = 6 in all subfigures.

**Fig 4:**
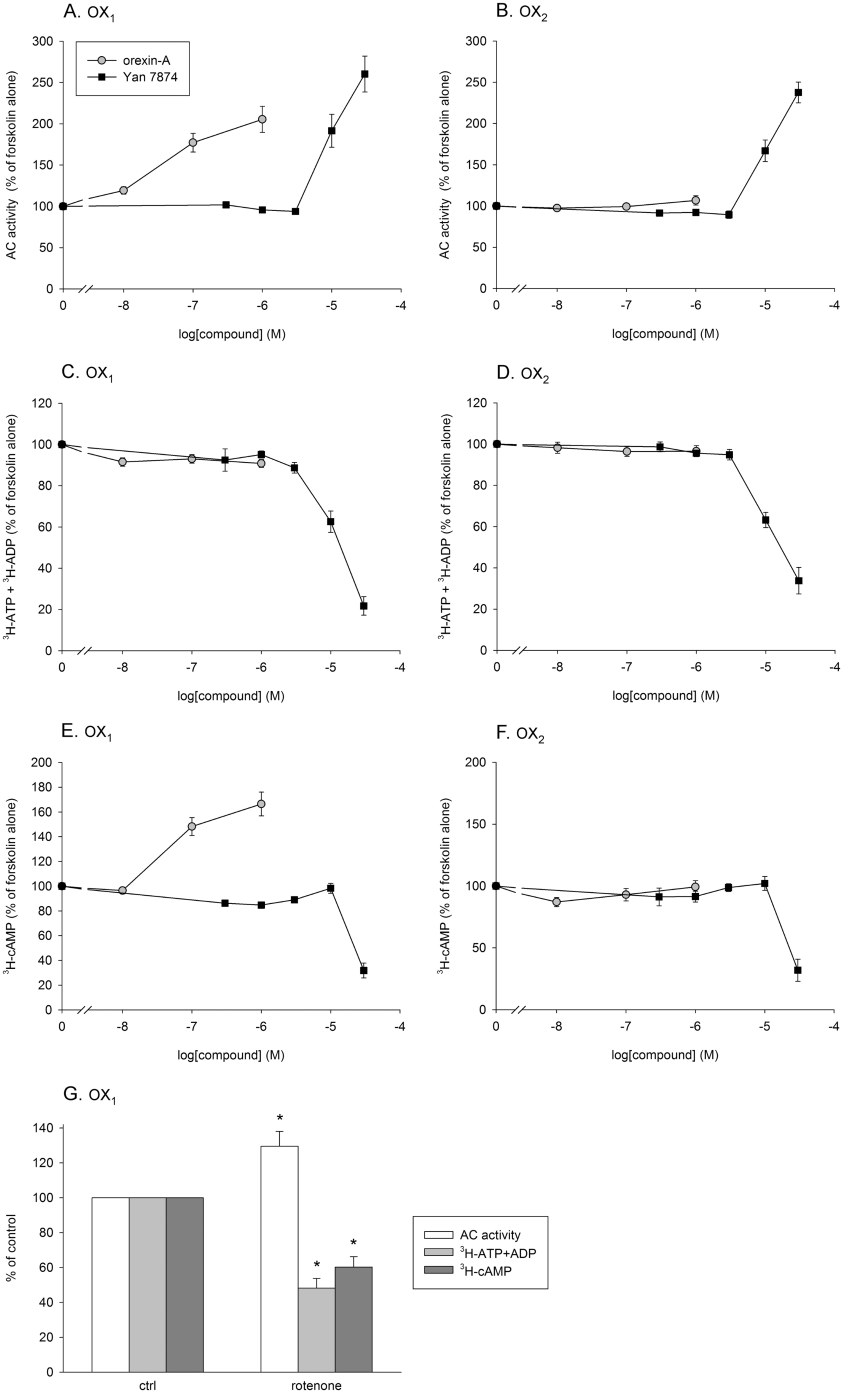
AC stimulation in cells treated with PTx as orexin-A and Yan 7874 concentration-response curves in orexin receptor-expressing CHO cells. (A–B) The apparent AC activity (^3^H-cAMP counts divided by ^3^H-ATP+ADP counts). (C–D) The counts in the ATP+ADP fraction from the ion exchange chromatography in PTx-treated cells. (E–F) "Pure" ^3^H-cAMP counts (not correlated to ^3^H-ATP+ADP counts). (G) The effect of rotenone (10 μM) on the apparent AC activity, ATP+ADP levels, and the "pure" ^3^H-cAMP counts. The experiment was performed only with CHO-hOX_1_ cells. The comparisons are to the control for each parameter. For all subfigures, the responses were normalized to the forskolin response (100%) separately for each independent sample before averaging. *N* = 5 for all subfigures.

All data are presented as mean ± SEM. Student's non-paired (Fig 3C) or paired (Fig 4G) two-tailed *t*-test with Bonferroni correction for multiple comparisons was used for statistical comparisons. *P* > 0.05 (*) was considered statistically significant. Microsoft Excel was used for all data visualizations and analyses including curve fitting, as described in [23].

## Results

### Ca^2+^ and phospholipase C

Ca^2+^ elevation and PLC activation are very pronounced responses for orexin receptor activation in CHO cells. Ca^2+^ elevation is primarily driven by inositol-1,4,5-trisphosphate (IP_3_) -independent Ca^2+^ influx and secondarily by PLC-mediated IP_3_-dependent Ca^2+^ release (reviewed in [9]); both responses are mediated by the G_q_-family proteins [22]. In the current study, orexin-A demonstrated strong, concentration-dependent responses for Ca^2+^ elevation (Fig 2A, 2C and 2D). Yan 7874, likewise, induced a strong and concentration-dependent Ca^2+^ elevation, but its solubility hindered reaching saturation (Fig 2); 30 μM Yan 7874 already contains 0.3% dimethyl sulfoxide (DMSO).

For PLC, concentration-dependent orexin-A responses were also observed (Fig 3A and 3B); the maximum responses for 10 min stimulation were over 10-fold the basal level (S1 Fig). Yan 7874 only caused a rather modest stimulation of PLC at 10 min stimulation time (Fig 3A and 3B). We increased the stimulation time to 30 min, in case the action of Yan 7874 might be slower than that of orexin-A. This increased the maximum orexin-A responses (S1 Fig); maximum Yan 7874 response was increased in the same degree as the orexin-A response, but its concentration-response curve became bell-shaped (Fig 3A and 3B).

We then assessed the specificity of the responses utilizing the non-selective orexin receptor antagonist TCS 1102 (10 μM). Only a minor fraction, approximately 30–40% of the Ca^2+^ and PLC responses to Yan 7874 were blocked by 10 μM TCS 1102 (Figs 2C, 2D and 3A–3C, and S2 Fig). In contrast, TCS 1102 fully blocked the response to 1 nM orexin-A (only tested for Ca^2+^; S2 Fig). While orexin-A responses were fully blocked by 1 μM of the specific G_q_ inhibitor UBO-QIC (not shown here; see [22], the responses to 10 μM Yan 7874 (only tested for PLC), were only blocked by approximately 40–50% (Fig 3C). We then tested CHO cells not expressing orexin receptors (ctrl cells) in the Ca^2+^ and PLC assays. In these cells, there was no response to orexin-A (not shown). In contrast, Yan 7874 produced a response that was indistinguishable from the orexin receptor-independent response in OX_1_- or OX_2_ receptor-expressing cells (Figs 2C, 2D and 3C). Furthermore, Yan 7874 response in ctrl CHO cells, as tested for PLC, was not inhibited by the orexin receptor antagonist TCS 1102 or the G_q_ inhibitor UBO-QIC (Fig 3C).

### Adenylyl cyclase

Adenylyl cyclase activity can be used to assess G_i_ and G_s_ protein coupling when certain precautions are taken [22–24]. When assessed in the PTx-treated CHO cells in the presence of a protein kinase C inhibitor, orexin-mediated AC stimulation should indicate the low potency G_s_-coupling. In OX_1_ receptor-expressing cells, significant AC stimulation was seen upon stimulation with orexin-A (Fig 4A). This response is much weaker for OX_2_ receptors [23], and this time we did not see any significant response to orexin-A (Fig 4B). Yan 7874, at concentrations above 10 μM, apparently stimulated AC activity both in OX_1_ and OX_2_ receptor-expressing cells (Fig 4A and 4B). The method is based on correlation of the cAMP counts and the ATP+ADP counts (see **Adenylyl cyclase activity** under Materials and methods); hence compounds that reduce the ATP+ADP counts may indicate a false increase in AC activity. Indeed, Yan 7874, starting from 10 μM, strongly reduced ATP+ADP counts (Fig 4C and 4D). When the cAMP counts were taken directly without any correlation to the ATP+ADP counts, there was no indication of AC stimulation by Yan 7874, while orexin-A still stimulated AC via OX_1_ receptors (Fig 4E and 4F).

AC inhibition in CTx-treated CHO cells in the presence of a PKC inhibitor should indicate G_i_-coupling [23, 24], though there also is some indication of G_q_ involvement in this response [22]. This is a high potency coupling for orexin receptors in these cells. Orexin-A induced marked AC inhibition in both OX_1_ and OX_2_ cells (S3 Fig). In contrast, we managed to see no significant inhibition by Yan 7874 at concentrations lower than 30 μM. At concentrations ≥ 10 μM, Yan 7874 reduced ATP+ADP counts in an equal degree as in PTx-treated cells (S3 Fig).

The decrease of ATP+ADP counts by Yan 7874 in this assay, although not a direct measure of cellular ATP levels, may indicate that ATP levels are drastically going down in the cells. An obvious cause for decrease of cellular ATP levels is mitochondrial uncoupling. We thus tested the complex I inhibitor rotenone (10 μM). Rotenone gave an apparent increase in the AC activity; however, this was associated with a decrease in cellular ATP+ADP counts and an actual decrease in cellular cAMP (Fig 4G). This indicates that the effect of Yan 7874 mimics mitochondrial uncoupling.

### Cell death

The studies of Ca^2+^ elevation (Yan 7874 response inhibited by only 30–40% by the orexin receptor antagonists TCS 1102 in orexin receptor-expressing cells, significant response in cells not expressing orexin receptor), PLC (Yan 7874 response inhibited by only 40–50% by TCS 1102 or the G_q_ inhibitor UBO-QIC in orexin receptor-expressing cells, significant wholly non-UBO-QIC-sensitive response in cells not expressing orexin receptor, bell-shaped Yan 7874 response at 30 min), and AC (dramatic reduction of ATP+ADP counts by Yan 7874) suggest that Yan 7874 displays at least significant off-target activity but that it also may be cytotoxic. We thus assessed the growth and viability of CHO-hOX_1_ and OX_2_ cells upon exposure to Yan 7874, first by simple visual examination. As observed 24 h after the treatment, cells treated with 10 and 30 μM Yan 7874 had lost their characteristic shape, and were, instead, rounded up and rich in vacuoles. Cell number appeared smaller which is at least partially due to increased cell detachment.

**Fig 5:**
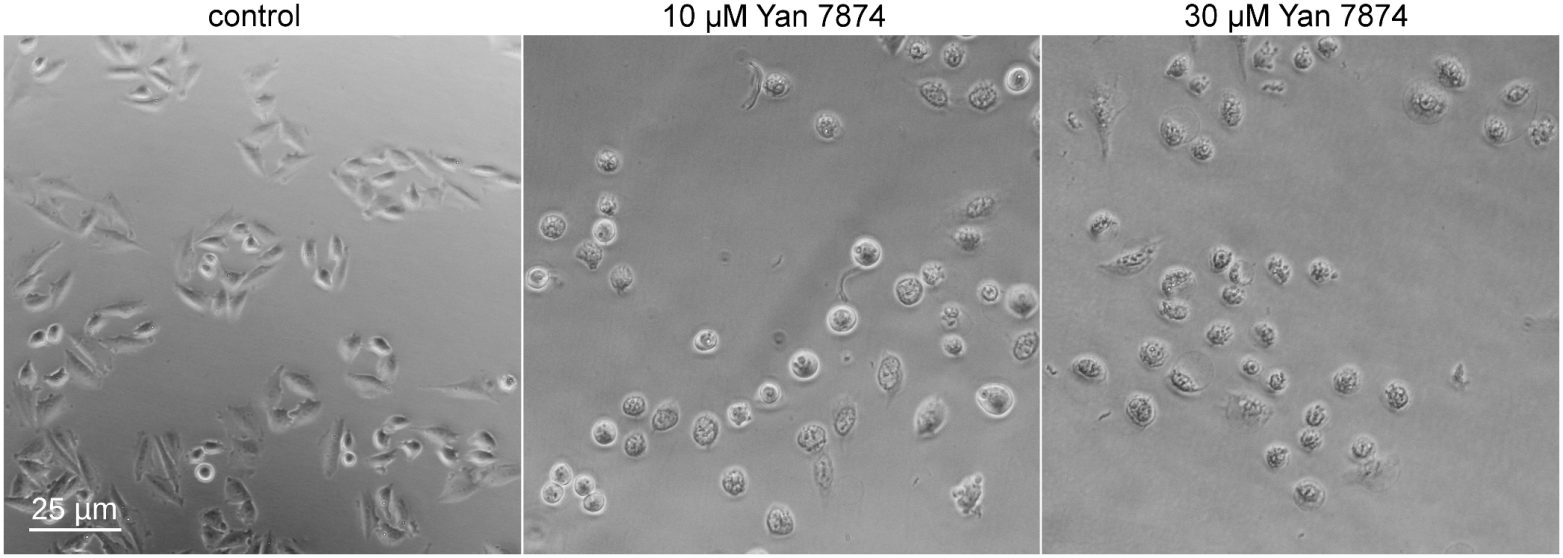
Morphological indications of cell death. Ctrl CHO cells (not expressing orexin receptor) were photographed under a phase contrast microscope after a 5-h treatment with 10 and 30 μM Yan 7874.

As we could not observe any inhibition of this process with TCS 1102, we went on to analyze control CHO cells (expressing no orexin receptors). These cells were also strongly affected by Yan 7874; already at the first time point after the treatment assessed, 5 h, marked morphological marks of cell demise, as described above, were seen for 10 and 30 μM Yan 7874 (Fig 5).

**Fig 6:**
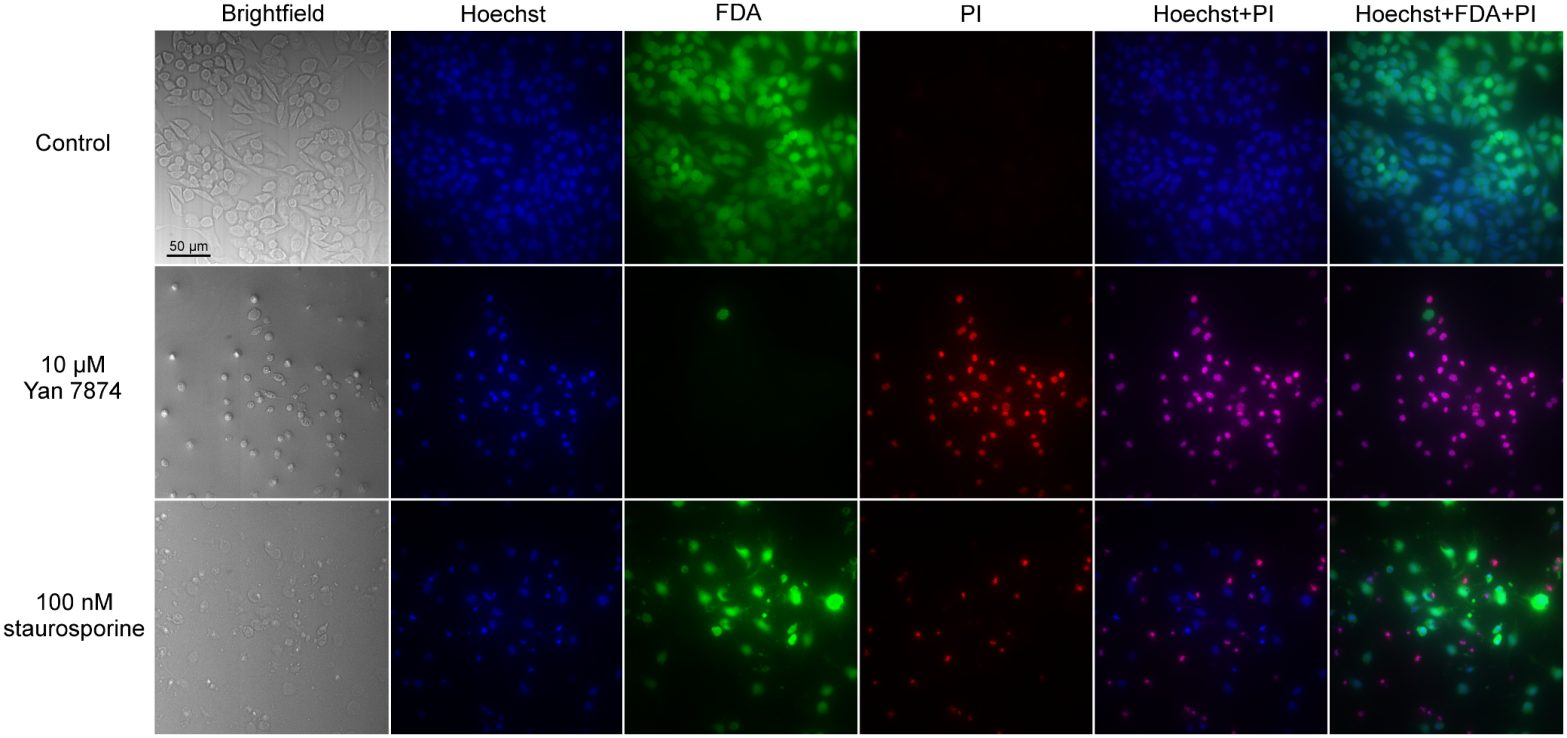
Different types of cell death in CHO cells. Ctrl CHO cells (not expressing orexin receptor), stained with Hoechst, FDA and PI, were photographed after 24 h of treatment with 10 μM Yan 7874 or 100 nM staurosporine. The brightfield images were taken with differential interference contrast; however, this effect is not very strong as 96-well plates were used.

We thus stained the cells with Hoechst, which is cell permeable and stains all nuclei; PI, which is non-permeable to viable and apoptotic cell membranes and thus only stains the nuclei of necrotic cells; and FDA, which is released and retained as a free, fluorescent form in the cytoplasm of viable cells only. Upon microscopic observation of these cells, we could observe that the fraction of viable cells (FDA-stained) was strongly reduced upon 24-h Yan 7874 treatment (Fig 6). Most nuclei in Yan 7874-treated cells did not appear pyknic (apoptotic), as assessed with Hoechst (Fig 6), but were stained with PI. In contrast, there were marked pyknic nuclei among cells treated with 100 nM staurosporine (Fig 6). These nuclei were partially PI-positive and partially PI-negative. This indicates that Yan 7874 induces mainly necrotic cell death, while staurosporine may first induce programmed cell death, including nuclear condensation and fragmentation, and the cells then continue to secondary necrosis (reviewed in [25]) as indicated by some PI-positivity. To verify that the cell death was not a CHO cell-specific response, we also assessed neuro-2a mouse neuroblastoma cells not expressing orexin receptors; these cells were also rapidly killed.

**Fig 7:**
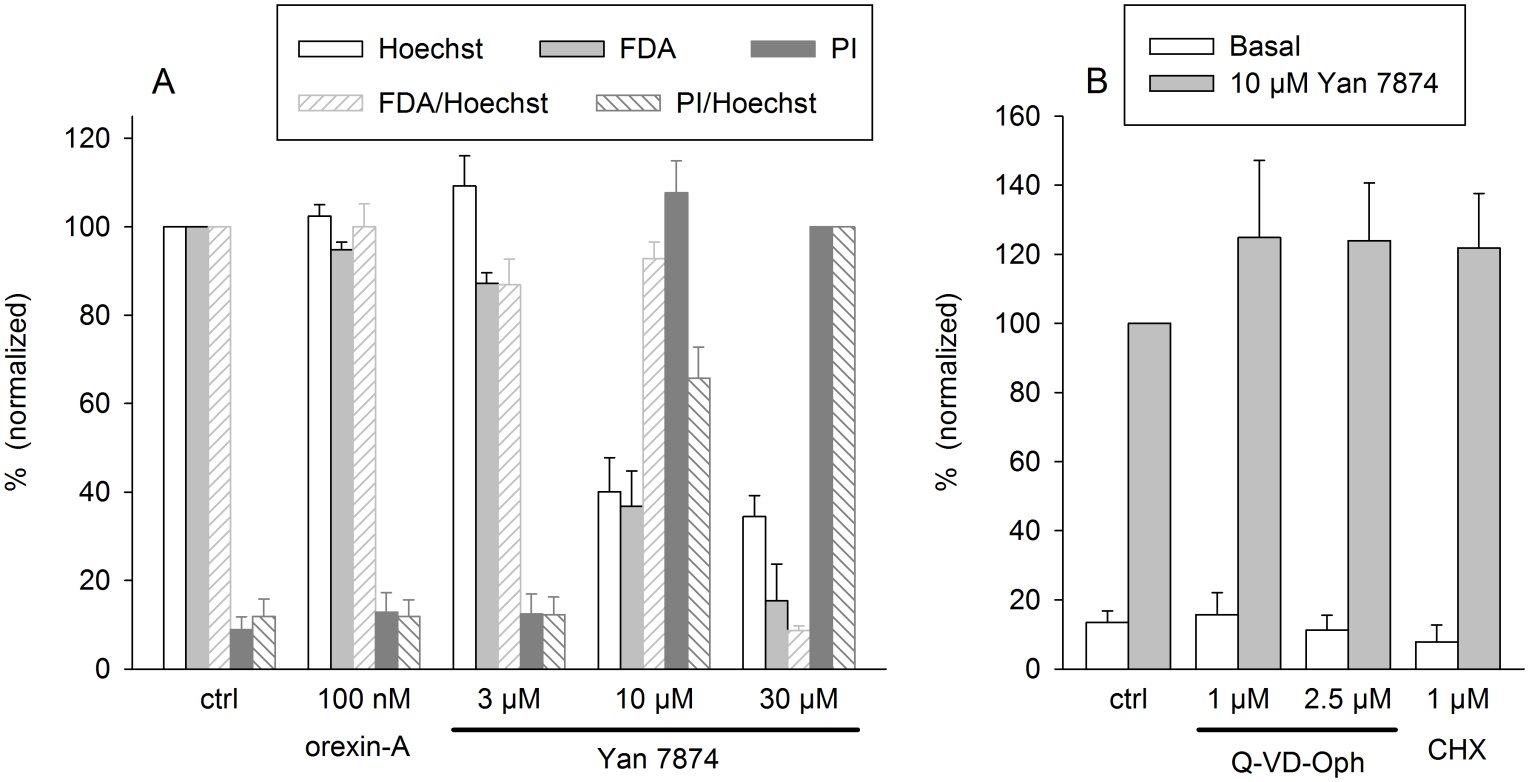
Quantitation of cell viability and demise upon Yan 7874 exposure in CHO cells. (A) Concentration-response relationship of Yan 7874. (B) The impact of the inhibition of caspases and protein synthesis. Ctrl CHO cells (not expressing orexin receptors) were cultured on 96-well plates for 24 after which they were treated with the compounds as indicated. After another 24 h, the cells were stained with Hoechst for total cell number, FDA for living cells, and PI for necrotic cells. (A) The values for total and living cells were normalized to the control, where essentially all cells are alive (see Fig 6) and thus the specific fluorescence is maximal. PI-stained cells could not be normalized in this way, since the specific PI fluorescence in control cells was essentially 0, and thus small deviations in the background subtraction would have introduced much noise. Therefore, the values were normalized to the PI fluorescence (or PI/Hoechst fluorescence ratio) in cells treated with 30 μM Yan 7874. "FDA/Hoechst" stands for the FDA reading divided by the Hoechst reading, indicating the proportion of living cells of all cells. "PI/Hoechst" similarly gives the proportion of necrotic/secondary necrotic cells among all cells. *N* = 6. (B) The effect of the pan-caspase inhibitor Q-VD-Oph and the protein synthesis inhibitor CHX on the response to Yan 7874 (10 μM). For the sake of clarity, only the PI/Hoechst fluorescence ratios, normalized to the control response to 10 μM Yan 7874 (in the absence of Q-VD-Oph or CHX), are shown. The ratios were always calculated separately for each independent sample before averaging. *N* = 8.

The fluorescence "viability and death parameters" were also quantitated on a plate reader after a 24-h treatment. Please observe that Hoechst fluorescent only gives the total cell number in this assay (**Cell viability and death** under Materials and methods) and that some release of free fluorescein from FDA may also occur in less viable cells. Yan 7874 was equally potent in reducing cell numbers and inducing cell death (Fig 7A). The solvent DMSO itself slightly reduced cell growth but did not induce cell death in the range used (S4 Fig). Yan 7874-induced cell death was not inhibited by fetal calf serum (results in the presence of serum are presented in all Figs 5–7). It also was insensitive to CHX, an inhibitor of protein synthesis, or Q-VD-Oph, a non-selective inhibitor of caspases (Fig 7B), supporting the notion that the cell demise occurred independent of cell death programs. This is in sharp contrast to the previously reported cell death upon orexin receptor activation, which occurs slowly by programmed cell death [26–29].

## Discussion

The results indicate that Yan 7874 is a weak partial orexin receptor agonist. It also induces non-specific effects in the same concentration range where orexin receptor-dependent responses are seen. The first observed off-target effects were a strong Ca^2+^ elevation and weak PLC activation. We also observed strong cellular toxicity in the same concentration range of Yan 7874. Ca^2+^ elevation is a very rapid response (in second scale), and also toxic effects are seen rather soon, starting from the apparent decrease in cellular ATP level. The results indicate a necrotic mechanism for the cell death, and the idea is supported by the rapid induction of cell death and the apparent decrease in the ATP level. A strong mitochondrial Ca^2+^ overloading is associated with necrotic death of many cell types (see e.g. [30]), and a similar rapid decrease in the apparent cellular ATP level is seen upon exposure to the mitochondrial toxin rotenone. However, we do not know in which—if any—way Ca^2+^ elevation or possible mitochondrial toxicity link to the death of the cells exposed to Yan 7874. One possibility is that the rapid intracellular Ca^2+^ elevation is a manifestation of membrane permeability increase, which might also cause a direct leak of cellular ATP, ADP and cAMP out of the cells. The verification of the exact mechanism of the cell death does not feel crucial, as the significant outcome of the study is that Yan 7874 cannot be recommended as a useful orexin receptor agonist tool. It is important to recognize that the induction of cell death by Yan 7874 is not anyhow linked to orexin receptors, as it is equally prominent in the absence of orexin receptors or orexin receptor activation. In addition, it lacks the hallmarks of orexin receptor-mediated cell death in CHO cells, e.g. cell death program (indicated by requirement of e.g. proteins synthesis) and sensitivity to fetal calf serum [27].

In the original publication (patent), which also is the only publication with this ligand thus far, Yan 7874 was shown to robustly activate an orexin-dependent reporter assay and to improve the narcoleptic phenotype of a orexin neuron-lacking rodent model, though the assay details are not quite clear [15]. It is hard to see any concurrence between the results of the current study and the study of Yanagisawa [15], but it must be noted that the outputs are also different. Based on the results of the current study we would predict a weak specific effect of Yan 7874 and rather significant toxicity, assuming that the compound was stable under physiological conditions. Based on our findings we do not think Yan 7874 can be recommended for orexin receptor studies. Nevertheless, we have recently shown that Yan 7874 can be utilized to reveal features required from orexin receptor agonists [31]. Furthermore, it is thinkable that the toxicity is associated with the structure of Yan 7874 in a way that it could be avoided in its analogues without compromising its ability to bind to and activate orexin receptors. Therefore, it might be worthwhile to assess some analogues of Yan 7874 as to determine whether they may display orexin receptor agonism without the toxicity. However, the efficacy of Yan 7874 also appears weak, and should also be clearly improved to make it useful as a drug or research tool.

## Supporting information

S1 Data The averaged values as presented in Figs 1–7 and S1–S4 Figs. (XLS)

S1 Fig The maximum stimulation obtained with orexin-A for PLC in CHO cells. The basal level is 1 and the orexin-A responses are given as times the basal level (as in Fig 3C and 3D). *N* = 8 for 10 min stimulation and 4 for 30 min stimulation. Basal levels at 10 and 30 min were not significantly different.
(PDF)

S2 Fig The effect of 10 μM TCS 1102 on Ca^2+^ responses to orexin-A and Yan 7874 in CHO cells. The results are gives as % of the maximum orexin-A response. *N* = 4.
(PDF)

S3 Fig AC measurements in CHO cells treated with CTx and stimulated with orexin-A and Yan 7874 in the presence of 10 μM forskolin. The results are given as % of the response to forskolin alone ("basal"). *N* = 4–6.
(PDF)

S4 Fig Cell viability upon exposure to different concentrations of the solvent DMSO. DMSO concentrations are in % (vol/vol). The experimental conditions are as in Fig 7A. *N* = 4.
(PDF)
