# Supplementary material for: Orexin receptor agonist Yan 7874 is a weak agonist of orexin/hypocretin receptors and shows orexin receptor-independent cytotoxicity
Source: PLoS One. 2017 Jun 2;12(6):e0178526. doi: 10.1371/journal.pone.0178526 (PMC5456073; doi:10.1371/journal.pone.0178526)
Supplement: S1 Fig — The basal level is 1 and the orexin-A responses are given as times the basal level (as in Fig 3C and 3D). N = 8 for 10 min stimulation and 4 for 30 min stimulation. Basal levels at 10 and 30 min were not significantly different. (PDF) [file pone.0178526.s002.pdf]

Ainoleena Turku, Maiju Rinne, Gustav Boije af Gennäs, Henri Xhaard, Dan Lindholm and Jyrki P. Kukkonen

Orexin receptor agonist Yan 7874 is a weak agonist of orexin/hypocretin receptors and shows orexin receptor-independent cytotoxicity

Plos One

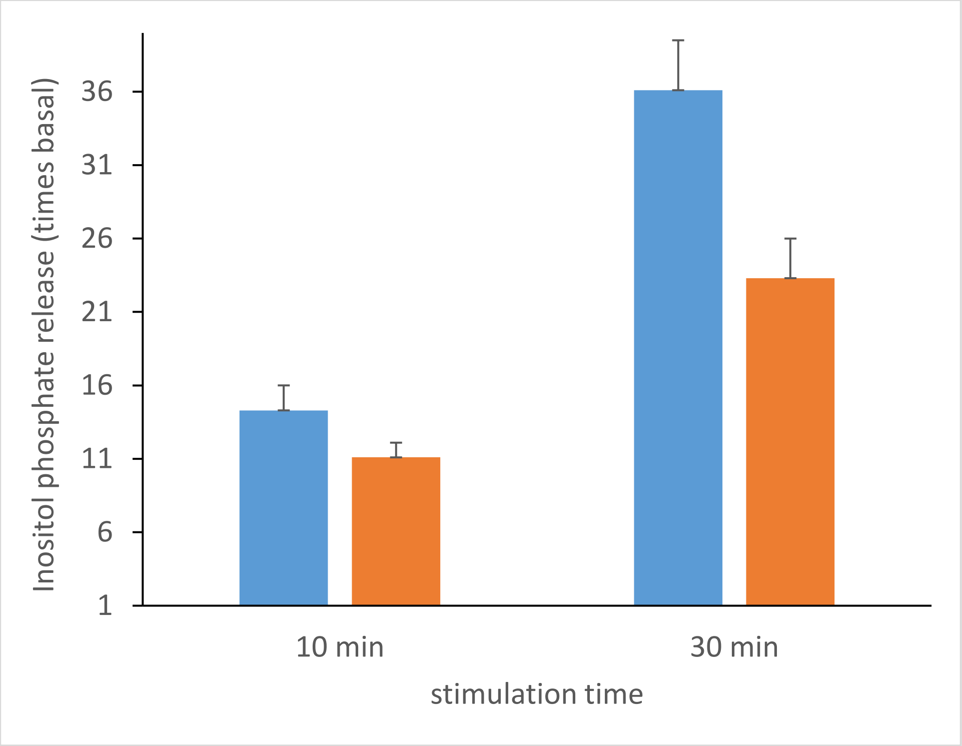

S1 Fig. The maximum stimulation obtained with orexin-A for PLC. The basal level is 1 and the orexin-A responses are given as times the basal level (as in Fig. 3C and D). *N* = 8 for 10 min stimulation and 4 for 30 min stimulation.
