# Supplementary material for: Orexin receptor agonist Yan 7874 is a weak agonist of orexin/hypocretin receptors and shows orexin receptor-independent cytotoxicity
Source: PLoS One. 2017 Jun 2;12(6):e0178526. doi: 10.1371/journal.pone.0178526 (PMC5456073; doi:10.1371/journal.pone.0178526)
Supplement: S2 Fig — The results are gives as % of the maximum orexin-A response. N = 4. (PDF) [file pone.0178526.s003.pdf]

Ainoleena Turku, Maiju Rinne, Gustav Boije af Gennäs, Henri Xhaard, Dan Lindholm and Jyrki P. Kukkonen

Orexin receptor agonist Yan 7874 is a weak agonist of orexin/hypocretin receptors and shows orexin receptor-independent cytotoxicity

Plos One

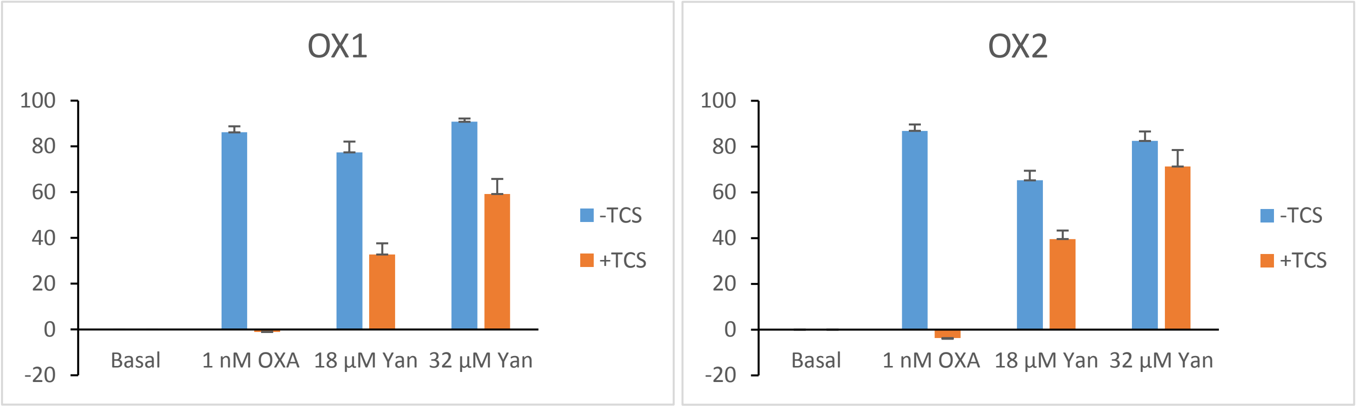

S2 Fig. The effect of 10 µM TCS 1102 ("+TCS") on Ca^{2+} responses to 1 nM orexin-A ("OXA") and 18 and 32 µM Yan 7874 ("Yan"). The results are gives as % of the maximum orexin-A response. $N = 4$.
