# Supplementary material for: Orexin receptor agonist Yan 7874 is a weak agonist of orexin/hypocretin receptors and shows orexin receptor-independent cytotoxicity
Source: PLoS One. 2017 Jun 2;12(6):e0178526. doi: 10.1371/journal.pone.0178526 (PMC5456073; doi:10.1371/journal.pone.0178526)
Supplement: S3 Fig — The results are given as % of the response to forskolin alone ("basal"). N = 4–6. (PDF) [file pone.0178526.s004.pdf]

Ainoleena Turku, Maiju Rinne, Gustav Boije af Gennäs, Henri Xhaard, Dan Lindholm and Jyrki P. Kukkonen

Orexin receptor agonist Yan 7874 is a weak agonist of orexin/hypocretin receptors and shows orexin receptor-independent cytotoxicity

Plos One

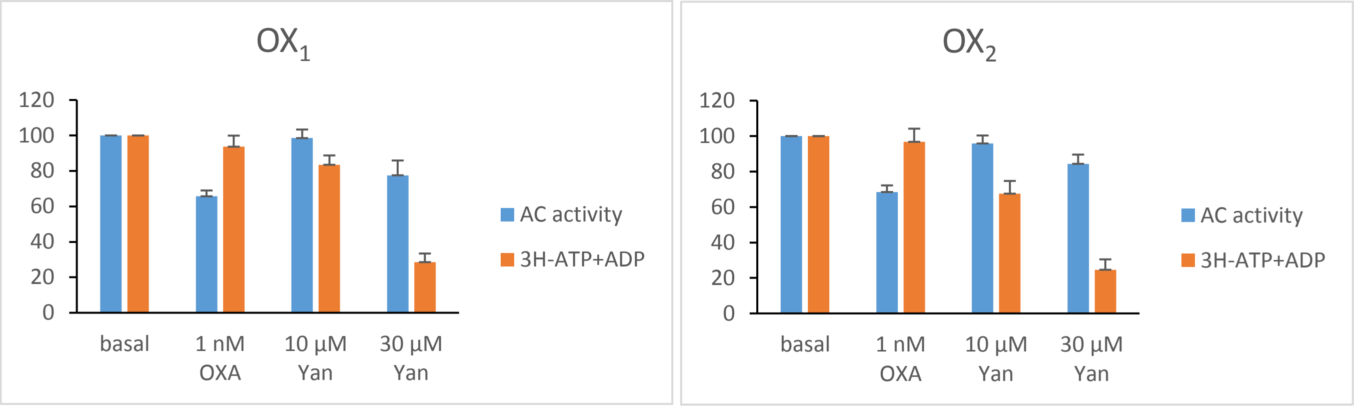

S3 Fig. **AC measurements in orexin receptor-expressing cells treated with CTx and stimulated with orexin-A ("OXA") and Yan 7874 ("Yan") in the presence of 10 µM forskolin.** The results are given as % of the response to forskolin alone ("basal"). *N* = 4–6.
