# Supplementary material for: Orexin receptor agonist Yan 7874 is a weak agonist of orexin/hypocretin receptors and shows orexin receptor-independent cytotoxicity
Source: PLoS One. 2017 Jun 2;12(6):e0178526. doi: 10.1371/journal.pone.0178526 (PMC5456073; doi:10.1371/journal.pone.0178526)
Supplement: S4 Fig — DMSO concentrations are in % (vol/vol). The experimental conditions are as in Fig 7A. N = 4. (PDF) [file pone.0178526.s005.pdf]

Ainoleena Turku, Maiju Rinne, Gustav Boije af Gennäs, Henri Xhaard, Dan Lindholm and Jyrki P. Kukkonen

Orexin receptor agonist Yan 7874 is a weak agonist of orexin/hypocretin receptors and shows orexin receptor-independent cytotoxicity

Plos One

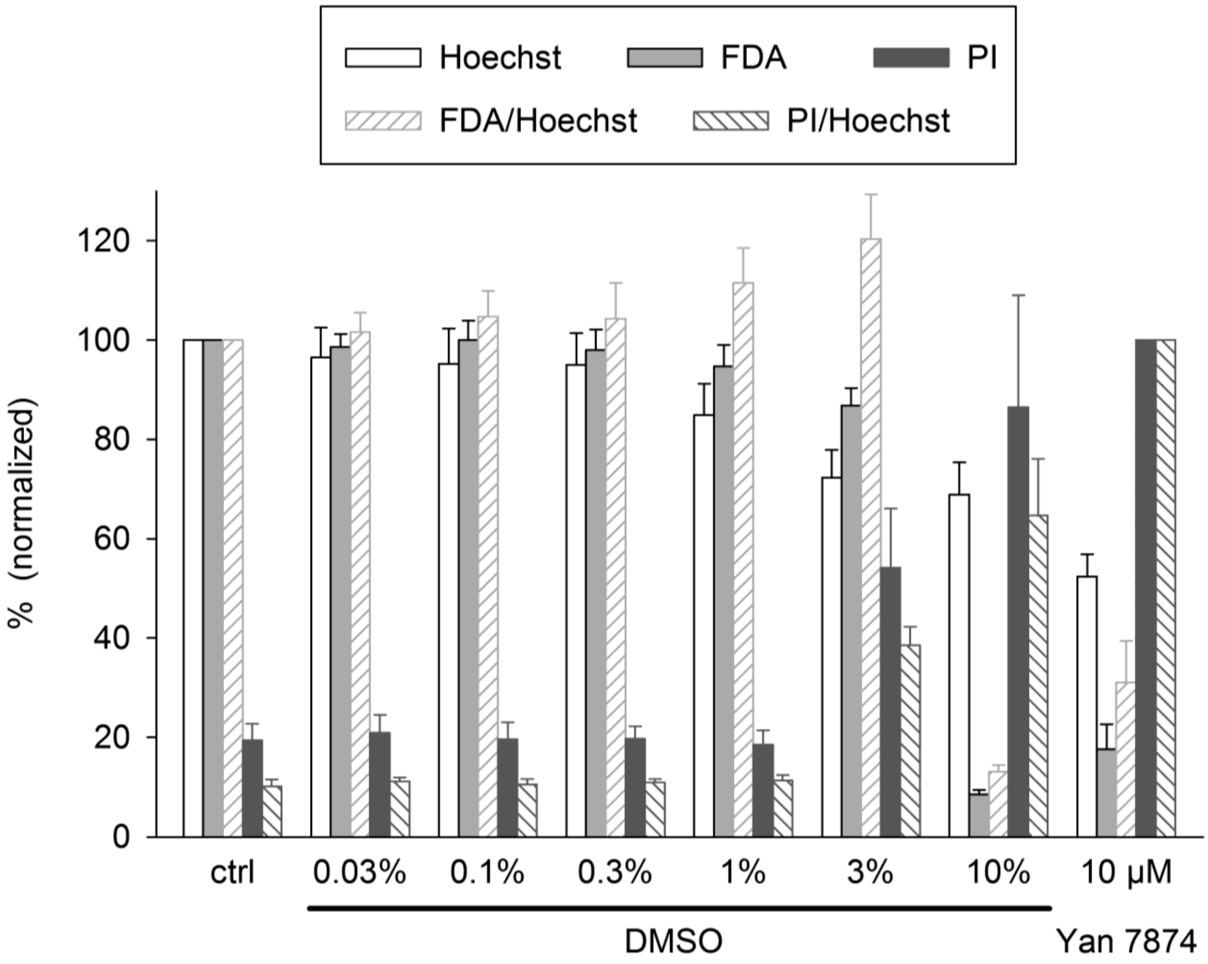

S4 Fig. Cell viability upon exposure to different concentrations of the solvent DMSO. DMSO concentrations are in % (vol/vol). The experimental conditions are as in Fig. 7A. $N = 4$.
